# Occurrence, Distribution, and Risk Assessment of Organophosphorus Pesticides in the Aquatic Environment of the Sele River Estuary, Southern Italy

**DOI:** 10.3390/toxics10070377

**Published:** 2022-07-07

**Authors:** Paolo Montuori, Elvira De Rosa, Fabiana Di Duca, Bruna De Simone, Stefano Scippa, Immacolata Russo, Michele Sorrentino, Pasquale Sarnacchiaro, Maria Triassi

**Affiliations:** 1 Department of Public Health, University “Federico II″, Via Sergio Pansini 5, 80131 Naples, Italy; elvira_derosa@libero.it (E.D.R.); fabianadiduca91@gmail.com (F.D.D.); desimonebruna7@gmail.com (B.D.S.); stefanoscippa923@gmail.com (S.S.); imrusso@unina.it (I.R.); sorrentinoemme@gmail.com (M.S.); triassi@unina.it (M.T.); 2 Department of Law and Economics, University “Federico II″, Via Cinthia 26, 80126 Naples, Italy; sarnacch@unina.it

**Keywords:** organophosphorus pesticides (OPPs), water dissolved phase, particulate phase, sediment, occurrence, riverine ecosystem, risk characterization, Sele River

## Abstract

The intensive use of organophosphorus pesticides (OPPs) causes concern among authorities in different countries, as many of them, remaining unchanged for a long time, pose a threat to environmental sustainability. This study assessed the spatio-temporal trends of nine OPPs in the water dissolved phase (WDP), suspended particulate matter (SPM), and sediment samples from the Sele River estuary, Southern Italy. Samples were collected in 10 sampling sites during four seasons. The highest levels were found at the mouth (mean value 28.25 ng L^−1^ as WDP + SPM) and then decreased moving southwards to the Mediterranean Sea. Moreover, highest concentrations were detected in the warm season (July) with a mean value of 27.52 ng L^−1^. The load contribution to the Mediterranean Sea was evaluated in about 61.5 kg year^−1^, showing that the river was an important source of OPPs through discharge into the sea. The risk assessment revealed that no high-risk indices for the general-case scenario were observed, but for the worst-case scenario, potential risks were associated with chlorpyrifos, pyrimifos-methyl, and parathion, suggesting that OPP contamination should not be neglected. This study makes up the first record of OPPs in the surface waters of the Sele River and provides helpful data as a starting point for future studies.

## 1. Introduction

The exponential growth of the world’s population since the beginning of the 21st century led to an increasing demand for food crops [1]. Consequently, among the many intense processes of agriculture, the use of pesticides is increasingly widespread, as their application to crops plays a key role in maintaining highly efficient agricultural productivity, leading to an improvement in the quality and an increase in the quantity of crops produced [2,3,4,5,6]. Around 20,000 tons of chemical pesticides are employed worldwide every year, out of which 24% is used in the United States and 45% in Europe [7]. However, the extensive use of pesticides in agricultural and urban areas not only affects the target organisms, but also poses a threat to the whole environment, including atmosphere, soil, groundwater, and surface water by flow, leaching, and pulverization processes, thus representing a potential concern for the ecosystem [8,9]. Several types of pesticides, including organophosphorus pesticides, neonicotinoids, organochlorine, carbamates, triazoles, amides, and so on, are applied annually to crops [10]. In particular, due to their high efficiency, broad spectrum and relatively short half-life in the environment, the organophosphorus pesticides (OPPs) are used as substitutes for organochlorine (OCPs) and carbamate pesticides, as they are considered a more environmentally safe alternative, accounting for more than 38% of the global pesticide market [11,12,13]. OPPs replaced OCPs when their use was banned in most developed countries after the Stockholm Convention [14,15,16]. In fact, compared to the OCPs, which belong to the class of persistent organic pollutants (POPs) and whose characteristics are persistence, toxicity, and long-range environmental transport [6,17], the OPPs are considered to be less persistent, due to their quick chemical degradation by air, sunlight, and water, and it is thought that they have less adverse impacts on the environment [10,18]. However, because of the massive annual applications of OPPs, they are constantly introduced in significant amounts to the environmental compartments, such as water, soil, sediment, and biota, via nonpoint (e.g., farming practices) or point (e.g., industrial discharges) sources [19], leading to the current levels of environmental pollution, especially in water systems [20,21,22]. According to the study by Wee et al. [19], a continuous increase in pesticide use is projected based on increasing atmospheric temperatures, so the ecological impacts of pesticides are likely to enhance in the future.

OPPs are synthetic compounds produced by reacting alcohols with phosphoric acid [5,23]. Referring to the chemical structures and functional groups, organophosphorus pesticides (OPPs) are phosphates, phosphoramides, or phosphorothioates, which typically contain P–O, P–N, or P–S bonds, respectively [9]. They are widely used worldwide as fungicides, herbicides, and insecticides for protecting against parasites, bacteria, and weeds, thereby reducing crop damages [24,25]. However, after application only a small percentage of them (~0.1%) act on target organisms [18,26], while the rest move into the environment through natural diffusion processes like surface runoff, wind or soil erosion, spray dispersion, and preferential water flow [10,27]. OPPs, by infiltrating and percolating through the structure of the soil, reach the surrounding water bodies and alter the nutrient content, influencing the microorganisms fundamental to the fertility of the soil [16,28,29].

As a result of their wide use, the application trend of OPPs raises many concerns all around the world; also because most of them are highly toxic [23,30], and the continuous release of these pesticides in the water ecosystem results in various physical and chemical effects such as bioaccumulation, which produces adverse effects ecologically and on the health of the population living in direct and/or indirect contact with pesticides [6,7,21]. In addition to humans, OPP’s toxicity poses a threat to aquatic organisms by causing immune depression, altering the organism’s immune function. Particularly, OPPs are known to exert toxicity towards fish species, altering the histopathology of internal organs and causing endocrine dysfunction as well as immune depression [16]. Moreover, in regard to chlorpyrifos, Wee et al. reported developmental toxicity (i.e., morphological abnormality), behavioral changes, oxidative stress, and immunotoxicity in the early life stages of zebrafish, and Huang et al. observed the necrosis of different tissues and organs (liver and kidney), and DNA toxicity to freshwater fish [1,19]. Furthermore, the coexistence of several pesticides in water has the potential to induce synergistic effects that increase toxicity and lead to ecological issues even at low levels [30]. Therefore, the quality of the surface water, which constitutes the largest source for drinking water treatment in most places, is compromised and could be a potential exposure pathway for humans [15,21,31].

The exposure to the chemical pesticides is extremely destructive for the flora, fauna, and the environment, and poses a danger to human health [7,32]. In fact, several pesticides, including OPPs, are considered as endocrine disrupting compounds (EDCs), because of their modes of action and mechanisms in endocrine system disruption [19,33]. Various types of health problems are due to the direct exposure, such as handling of pesticides or pesticide residues present in the food stuffs [25,34]. Acute OPP toxicity, both in humans and in animals, is caused by inhibition of acetylcholinesterase (AChE), resulting in an overstimulation of nicotinic and muscarinic receptors, which triggers a cholinergic crisis associated with respiratory problems [35,36]. Karami-Mohajeri et al. reported that every year more than three million poisoning cases by OPPs and approximately 200,000 mortalities are recorded [37]. Consequently, on the basis of epidemiological data, they were recently included on the list of banned substances in the European Union [38,39] due to their proven toxicity [40].

Pesticides migrate from crop terrains, via adhesion to soil particles, to agricultural drainages ditches, allowing them to reach aquatic ecosystems [19,41]. Moreover, the occurrence of OPPs in aquatic systems not only affects aquatic plants, but it also poses a threat for aquatic organisms [34,42,43].

In this context, it is necessary to investigate the spatial and temporal occurrence and ecological risks of these chemicals in water resources to ensure the safety of aquatic organisms and humans. In fact, OPP pollution can be a serious problem because these chemicals, being vulnerable to natural processes such as volatilization, adsorption, oxidation, biodegradation, and hydrolysis, can form degradation products that exhibit higher toxicity, persistence, and stronger endocrine disrupting effects than parent compounds [19,24,44,45].

The Sele River flows through an alluvial-coastal plain, called the Sele River Plain, extended about 400 km^2^ and characterized by rich agricultural productivity and intense industrial activity [46,47,48,49]. These activities involve a massive use of pesticides and fertilizers, which can impair water quality. Particularly, the most used OPPs in the Sele River Plain were chlorpyrifos and dimethoate, with about 27 and 4 tons sold per year, respectively [50]. Many studies reported a high predominance of chlorpyrifos and dimethoate in several areas of southern Europe [6,51,52], including Italy [53,54,55], which led to them being identified as hazardous substances for water biodiversity in Mediterranean ecosystems [51,56,57]. Therefore, according to the last agriculture census by ISTAT (2010), in line also with the current European regulations, such as the Water Framework Directive (WFD) [58], and considering the current bibliography [3,5,10,16,23,27,30,34,59], the OPPs investigated in the study area were: diazinon, dimethoate, malathion, chlorpyrifos, pirimiphos-methyl, fenitrothion, methidathion, tolclofos-methyl, and parathion (Table 1). The WFD aims at achieving a good ecological status for all European water bodies and provided an environmental quality standard (EQS) that must be met for identified priority hazardous substances [57].

The literature lacks data on the occurrence of OPP in the Sele River estuary and its environmental impacts on the Tyrrhenian Sea (Central Mediterranean Sea). Therefore, this study aims to assess the concentrations of OPPs in the Sele River estuary, Southern Italy, and their environmental impact on the Mediterranean Sea. Particularly, this study has the purpose of (i) evaluating the OPP levels in the Sele River estuary and surrounding aquatic environment from the estuary towards the Central Mediterranean Sea; (ii) estimating their distribution between water dissolved phase, suspended particulate matter, and sediment; (iii) defining their spatial distribution and temporal trends in the study area; (iv) estimating the OPP inputs from the river into the Central Mediterranean Sea; (v) assessing the potential environmental impact of OPPs from the Sele River system and its estuary on the Mediterranean Sea, Southern Italy. To the best of our knowledge, there are no previous studies that evaluated the loads of OPPs from the Sele River and its environmental impact on the Mediterranean Sea.

## 2. Materials and Methods

### 2.1. Study Area

The Sele River is one of the most important waterways of the Campania Region, with a length of 64 km, a drainage basin of 3235 km^2^, and an annual mean flow rate of 69 m^3^/s. In terms of average water volume, it is the second river of the region and the South of Italy, behind the Volturno River, and it is a tributary of the Tyrrhenian Sea [49,60]. The river rises on the southeastern slopes of Monte Paflagone, near the Caposele in the province of Avellino. On its way to the sea, the river Sele receives at Contusi Terme the Tanagro, its main tributary, and near Ponte Barizzo, it receives the Calore Lucano, another important tributary. At Eboli, the river enters the floodplain known as the Sele River Plain, embedded in the natural reserve of Foce Sele-Tanagro [61]. The plain is bordered towards the sea by a straight sandy coast, which extends between the towns of Salerno and Agropoli, and landward by the Lattari and Picentini Mountains (to the north and northwest, respectively), and by the Alburni Mountains and Cilento Promontory (to the southeast) [46,47,48].

Thanks to the numerous reclamations carried out on the territory, the Sele River Plain was able, over time, to become fertile and luxuriant soil for both agriculture and breeding. To date, the plain is a prolific reality, rich in arable fields processed through the best farming techniques. Furthermore, the industrial activities are numerous, but the emissions of wastes with a high load of chemicals, including OPPs, imply negative effects on the ecosystem, causing health problems and environmental deterioration [43,49].

### 2.2. Sampling

**Figure 1:**
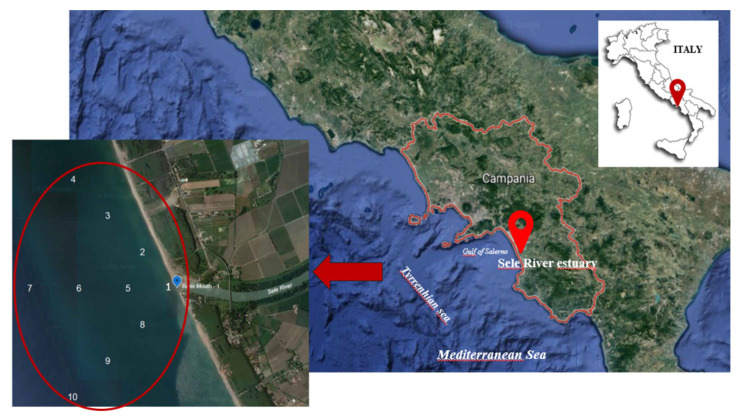
Map of the study area and sampling locations in the Sele River estuary, Southern Italy (Sampling sites: 1—River mouth; 2—Nord estuary at 500 m; 3—Nord estuary at 1000 m; 4—Nord estuary at 1500 m; 5—West estuary at 500 m; 6—West estuary at 1000 m; 7—West estuary at 1500 m; 8—South estuary at 500 m; 9—South estuary at 1000 m; 10—South estuary at 1500 m).

Between 2020 and 2021, water and sediment samples were collected in four different seasons to assess the spatial and temporal trends of OPP concentrations in the Sele River estuary. The sampling was carried out in July and November 2020, as well as in February and April 2021. The location of the sampling stations is displayed in Figure 1 and the characteristics of each site are detailed in Table 2, Table 3 and Table 4. Samples were collected at 10 locations: one at the river mouth and the other nine at different distances from the mouth, i.e., 500 m, 1000 m, and 1500 m to the north, south, and west.

Surface water samples were taken at approximately 20 cm of the river’s depth by using 2.5-litre dark glass bottles [55,63,64,65]. After collection, water samples were taken to the laboratory as soon as possible and stored in darkness at 4 °C until analysis, which was carried out within two days. Water samples were passed through a glass fibre filter (47 mm × 0.7 µm; Whatman, Maidstone, UK) to obtain the water dissolved phase (WDP) and the suspended particulate matter (SPM) fractions. Filters (SPM) were kept in the dark at −20 °C until analysis, while for filtrates (WDP), the analyses were carried out within 6 h of sampling. The sediment (SED) samples (0–5 cm) were collected during the spring season (April 2021) at the 10 sampling stations. The samples were collected using a Van Veen grab sampler and immediately transferred into aluminium containers and stored at −18 °C within 1 h after sampling.

### 2.3. Extraction Procedure and Clean-Up

In order to perform the OPP extraction, the methodology proposed by Cruzeiro et al. was used [8]. Briefly, WDP samples (500 mL) were adjusted to pH~7, if necessary, using 1:1 (*v*/*v*) sulfuric acid, and preconcentrated using SPE Oasis HLB cartridges (6 mL, 500 mg; Waters, Milford, MA, USA), previously preconditioned with 5 mL of ethyl acetate (EtOAc), 5 mL of methanol (MetOH), and 2.5 mL of deionized water. The extracts were eluted with 6 mL of EtOAc, evaporated to dryness, and reconstituted in hexane for analysis by GC-MS. The OPP extraction from the SPM samples was performed using an ultrasonic bath, soaking the filters in 3 mL of EtOAc for 8 min. Therefore, the extracts were evaporated to dryness under a gentle stream of N_2_ and reconstituted in hexane for analysis by GC-MS. For sediment, 5 g aliquots were used. After drying (60 °C) and sieving (250 μm), the OPPs were extracted from SED samples three times by sonication for 15 min using 15 mL of a dichloromethane/methanol (1:1) mixture. The extracts were centrifuged, concentrated and reconstituted in hexane for instrumental analysis [53,54,55].

Semi-volatile organic compound (SVOC) surrogate standard (2-fluorobiphenyl, nitrobenzene-d_5_, p-terphenyl-d_14_, 2-fluorophenol, phenol-d_5_, 2,4,6-tribromophenol) and SVOC internal standard (acenaphtene-d_10_, crysene-d_12_, 1,4-dichlorobenzene-d_4_, naphtalene-d_8_, perylene-d_12_, phenantrene-d_10_) mixtures were used as surrogate and internal standards, respectively. To check the method, all samples were analysed in triplicate. The SVOC surrogate standard was added to all samples before extraction to monitor the efficiency of the analytical procedure and the SVOC internal standard was added into each pretreated sample just before the injection in order to monitor the substrate effects in instrumental analysis and to quantify the target analytes on the basis of the internal standard method.

### 2.4. Instrumental Analysis

OPP identification and quantification were performed by a TRACE^TM^1310 Gas Chromatograph coupled to a ISQ^TM^7000 Single Quadrupole Mass Spectrometer (GC-MS, Thermo Scientific, Waltham, MA, USA), and equipped with a TG-5MS capillary column (30 m × 0.25 mm i.d. × 0.25 μm film thickness). The helium was the carrier gas (constant flow rate of 1 mL/min). Splitless mode was selected to inject 1 μL sample volume. The injector and detector temperatures were set to 250 and 280 °C, respectively. The oven temperature was programmed as follows: 35 °C for 3 min, increasing to 100 °C at 25 °C min^−1^ (kept for 4 min), to 280 °C at 30 °C min^−1^ (kept for 4 min), and finally, to 320 °C at 10 °C min^−1^ (kept for 1 min).

OPP quantification was made using a five-point calibration curve for the investigated chemicals (purchased from Agilent Ultra Scientific, Bologna, Italy) (R^2^ > 0,995). OPPs were identified according to their retention time and transition ions (quantitation ions and qualification ions). According to current European regulations, such as the Water Framework Directive, the OPPs investigated were: diazinon, dimethoate, malathion, chlorpyrifos, pirimiphos-methyl, fenitrothion, methidathion, tolclofos-methyl, and parathion [58].

### 2.5. Quality Assurance and Quality Control

Procedural blanks were processed in the same manner as real samples and evaluated with each set of water samples. The limits of detection (LODs) and quantification (LOQs) were estimated as three and ten times, respectively, the signal /noise (S/N) level for individual analyte. The performance of the method was daily checked to verify that LODs and LOQs were achieved, to confirm the absence of contamination and to ensure data quality. So, for each batch of the ten samples analysed, a procedural blank and a spiked recovery sample obtained by spiking at the low level, were routinely extracted and analysed under the same conditions as the ordinary samples. LODs of the detected analytes were in the range of 0.030–0.063 ng L^−1^ for water and suspended particulate samples and 0.039–0.135 ng g^−1^ for sediment samples. Data below the LOD were indicated as n.d. (not detected). The LOQs of the detected analytes were in the range of 0.100–0.210 ng L^−1^ for water and suspended particulate samples and 0.130–0.450 ng g^−1^ for sediment samples. Results of the OPPs were corrected for surrogate recoveries, both in standards and samples, which were all between 70 and 130%, satisfying quality control requirements. Calibration plots had a satisfactory linear regression coefficient (R^2^ > 0.995) for all analytes.

### 2.6. OPP Input Estimation and Statistical Analysis

The annual OPP discharge into the Mediterranean Sea was evaluated according to the UNEP guidelines [66], using the flow-averaged mean amount, obtained as the product of instantaneous concentration and the daily average water flow discharge for each sampling event and corrected by the total water load for the sampling period (July 2020–April 2021) [67]. River flow data were found in the database of the Autorità di Bacino Distrettuale dell’Appennino Meridionale Sede Basilicata.

Data obtained, expressed as the pesticide average concentration ± standard deviation (SD), were analysed using IBM SPSS (vs. 27) statistical software program (SPSS Inc., Chicago, IL, USA). Results with *p* values ≤ 0.05 were considered to be of statistical significance.

For principal component analysis (PCA), the selection of the components to retain is based on three main criteria: the Eigenvalue one criterion, according to the first components with Eigenvalues > 1 are considered; amount of explained variance, according to which the factors chosen should explain at least 70–80% of the variance; scree plot, (graphical method) which provides that the factors until a break in the graph are chosen.

### 2.7. Risk Assessment

The risk assessment associated with the detected analytes was based on risk quotient (*RQ*) values [19,34,55]. For each individual contaminant found in WDP, the *RQ*_(*contaminant)*_ was calculated using the ratio of the mean or maximum measured environmental concentration (*MEC*) to the predicted no-effect concentration (*PNEC*), as shown by the Equation (1) [3,32].

(1) $$RQ_{\left(contaminant\right)}=\frac{\;MEC}{PNEC}$$

The mean concentration detected was used to evaluate the *RQ_m_* (general-case scenario), while for the worst-case scenario, the *RQ_ex_* using the maximum concentration detected was calculated [10,16,27]. In addition, the risk quotient of the OPP mixture (*RQ_mix_*) was evaluated as the sum of individual *RQ_m_* values of each OPP [19,68].

To evaluate the *PNEC* for chronic toxicity, the no observed effect concentration (*NOEC*) was used. In particular, the *PNEC* was evaluated by dividing the toxicity value by an assessment factor (*AF*), as shown in Equation (2). The *AF* may be 10, 50, or 100 when three, two, or one trophic levels of *NOEC* values, respectively, are available [5,23].

(2) $$PNEC_{chronic}=\frac{NOEC}{AF}$$

In absence of chronic toxicity data, the effective concentration of 50% (*EC*_50_) or the lethal concentration of 50% (*LC*_50_) observed in sensitive species in aquatic ecosystems from the Pesticide Properties DataBase (PPDB, 2021) [62] were used to calculate the *PNEC* for *acute* toxicity, according to the Equation (3). In this case, the *AF* is 1000 [16,30,60].

(3) $$PNEC_{acute}=\frac{LC_{50\;}\left(or\;EC_{50}\right)}{1000}$$

Long-term data (*NOEC*) are preferred to short-term data (*LC*_50_ or *EC*_50_) because *AF* applied for long-term tests are smaller, and so the uncertainty related to extrapolation from laboratory data to the natural environment is reduced [68].

High *MEC* and/or low *PNEC* are indicators of a higher ecological risk. To best reflect ecological systems, native aquatic organisms from three trophic levels (fish, aquatic invertebrates, and algae) were considered in this study. Four risk levels were identified based on the value of *RQ*: negligible risk (*RQ* < 0.01), low risk (0.01 < *RQ* < 0.1), medium risk (0.1 < *RQ* < 1), and high risk (*RQ* > 1). Therefore, an *RQ* > 1 suggests high ecological risk and the environmental risk could not be excluded [22,32,34,69,70,71].

## 3. Results and Discussions

### 3.1. Occurrence of OPPs in WDP, SPM, and SED

**Figure 2:**
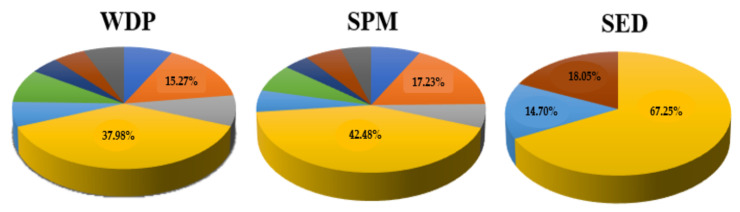
Total OPP amount % detected for each analyte in WDP (water dissolved phase), SPM (suspended particulate matter), and SED (sediment) of the Sele River, Southern Italy in 10 sampling sites and four seasons.

The results obtained from the analyses of the WDP, SPM (detailed in Tables S1 and S2, respectively), and SED samples are indicated in Table 2, Table 3 and Table 4, respectively. Particularly, the total amount of OPPs detected in WDP (Table 2 and Table S1) ranged from 1.02 ± 0.20 ng L^−1^ (Station 7, 1500 m westward—February) to 43.24 ± 1.06 ng L^−1^ (Station 1, River Mouth—July), with a mean value of 9.82 ± 9.36 ng L^−1^. In detail, the concentrations found for the individual analytes were in the range of n.d.—3.17 ng L^−1^ (mean value of 0.74 ± 0.92 ng L^−1^) for diazinon, n.d.—10.08 ng L^−1^ (mean value 1.50 ± 2.06 ng L^−1^) for dimethoate, n.d.—3.24 ng L^−1^ (mean value 0.80 ± 0.87 ng L^−1^) for malathion, 0.17–14.08 ng L^−1^ (mean value 3.74 ± 3.41 ng L^−1^) for chlorpyrifos, n.d.—3.03 ng L^−1^ (mean value 0.64 ± 0.58 ng L^−1^) for pirimiphos-methyl, n.d.—4.92 ng L^−1^ (mean value 0.87 ± 0.90 ng L^−1^) for fenitrothion, n.d.—1.21 ng L^−1^ (mean value 0.44 ± 0.35 ng L^−1^) for methidathion, n.d.—4.03 ng L^−1^ (mean value 0.48 ± 0.87 ng L^−1^) for tolclofos-methyl, and n.d.—2.83 ng L^−1^ (mean value 0.62 ± 0.56 ng L^−1^) for parathion. The maximum concentrations detected in WDP were 14.08 ± 0.73 ng L^−1^ for chlorpyrifos and 10.08 ± 0.68 ng L^−1^ for dimethoate. In fact, chlorpyrifos and dimethoate were the most ubiquitous compounds among all analysed chemicals, as they were detected in almost all WDP samples with a contribution to total OPP concentrations of 37.98 and 15.27%, respectively (Figure 2). In addition, fenitrothion, malathion, diazinon, and pirimiphos-methyl were found in medium/low concentrations, contributing 8.85, 8.13, 7.52, and 6.54%, respectively, to the total amount of OPPs. The results were in agreement with the above, namely that the two most sold OPPs in the area of interest were chlorpyrifos and dimethoate [50]. However, since 1 April 2020, the European Union (EU) banned the marketing of pesticides containing chlorpyrifos, due to its potential genotoxicity and developmental neurotoxicity [39], and also because, as stated by Mit et al., the mechanisms of the low-dose effects of this substance as single chemicals and in a mixture are still unclear [72]. Therefore, the highest concentrations detected in WDP for chlorpyrifos were presumably due to its frequent use in the past for multiple applications, including agriculture; just consider that in 2016 it was one of the most frequently detected pesticides in fruit and vegetable samples taken in several southern European countries [51,65]. In addition, even if chlorpyrifos is moderately persistent in soil but not in water systems, the use of chlorpyrifos outside the European Union remains significant, so it still enters aquatic environments and, therefore, spreads worldwide via sea currents [73]. Furthermore, the considerable concentrations of dimethoate detected in WDP are probably due to its chemical and physical properties, such as its high-water solubility (25,900 mg L^−1^) and hydrophilic nature (Log*K_ow_* = 0.78), as indicated in Table 1 [62,63,64,65,66,67,68,69]. Additionally, on dimethoate, the EU issued a non-renewal report in 2019, but it is still authorized at national levels and used in many European and non-European countries, mainly on vegetable crops and in olive oil production [38,70,71]. In addition, the presence of tolclofos-methyl, diazinon, pirimiphos-methyl, and malathion could probably be due to their known past use in crop protection [72]. Compared to previous studies, the concentrations found in the WDP samples of the Sele River were higher than those found in the dissolved phase of the Ebro River (Spain) by Gòmez-Gutiérrez et al. [65] for diazinon (0.2–1.7 ng L^−1^), but lower than those detected for chlorpyrifos (n.d.—42.5 ng L^−1^). In addition, the concentrations found in the Sele River were higher than those of Mai et al. [73], whose detected concentrations ranged from n.d. to 0.046 ng L^−1^ for diazinon and n.d. to 0.045 ng L^−1^ for dimethoate in the dissolved phase of the North Sea (Germany). Cruzeiro et al. [8], on the contrary, noticed higher levels in the dissolved phase of the Tagus River (Spain) for chlorpyrifos (mean value 96.21 ng L^−1^), diazinon (mean value 106.31 ng L^−1^), dimethoate (mean value 155.12 ng L^−1^), fenitrothion (mean value 228.05 ng L^−1^), malathion (mean value 134.06 ng L^−1^), and parathion (mean value 23.76 ng L^−1^) than those detected in this study. In addition, higher diazinon amounts (mean value 32.8 ± 65.2 ng L^−1^) were found in the dissolved phase of the Linggi River (Malaysia) by Zainuddin et al. [16], but lower (mean value 0.50 ng L^−1^) than that of the Wenyu River (China) by Zhang et al. [23].

In Table 3, the OPP concentration ranges detected in SPM samples from the Sele River estuary during the sampling campaigns are shown (detailed in Table S2). The total amount of OPPs detected ranged from n.d. to 20.37 ± 0.23 ng L^−1^ (1571.91 ± 47.81 ng g^−1^ dry weight (dw)) at Station 1, River Mouth, with a mean value of 3.74 ± 4.88 ng L^−1^ (324.14 ± 447.39 ng g^−1^ dw). In detail, the ranges of concentrations found for the individual analytes were n.d.—1.75 ng L^−1^ (mean value 0.28 ± 0.51 ng L^−1^) for diazinon, n.d.—4.11 ng L^−1^ (mean value 0.64 ± 1.00 ng L^−1^) for dimethoate, n.d.—1.40 ng L^−1^ (mean value 0.23 ± 0.39 ng L^−1^) for malathion, n.d.—6.58 ng L^−1^ (mean value 1.59 ± 1.86 ng L^−1^) for chlorpyrifos, n.d.—1.54 ng L^−1^ (mean value 0.21 ± 0.36 ng L^−1^) for pirimiphos-methyl, n.d.—2.38 ng L^−1^ (mean value 0.26 ± 0.53 ng L^−1^) for fenitrothion, n.d.—0.72 ng L^−1^ (mean value 0.15 ± 0.23 ng L^−1^) for methidathion, n.d.—2.64 ng L^−1^ (mean value 0.21 ± 0.54 ng L^−1^) for tolclofos-methyl, and n.d.—0.97 ng L^−1^ (mean value 0.17 ± 0.30 ng L^−1^) for parathion. The maximum concentrations detected in SPM were 6.58 ± 0.37 ng L^−1^ for chlorpyrifos and 4.11 ± 0.16 ng L^−1^ for dimethoate. As for WDP, chlorpyrifos and dimethoate were the most ubiquitous compounds among all analysed chemicals, with a contribution to total OPP concentrations of 42.48 and 17.23%, respectively, followed by diazinon, fenitrothion, and malathion, which were found in medium/low concentrations with a percentage contribution of 7.50, 7.08, and 6.05%, respectively, to the total OPP amount (Figure 2). High levels of chlorpyrifos may be due to the high adsorption affinity suspended particulate matter (K_oc_ = 5010 mL g^−1^), while for dimethoate, considering its very low adsorption affinity (K_oc_ = 15.8 mL g^−1^), it could be explained by its moderate/high mobility, its susceptibility to leaching out from the soil to the surface water [69,74,75] but also its massive use in agricultural practices as a substitute for other organophosphorus pesticides, such as azinphos-methyl, chlorfenvinphos, ethion, and fenthion, which were banned in Europe [76]. Dimethoate sorption kinetics followed a pseudo second-order model with an initial faster sorption due to partition into soil organic matter and clay surfaces, and a slower sorption by gradual diffusion of the molecule into soil micropores [75]. For SPM, it is difficult to compare with other previous studies because the concentrations were evaluated on the water matrix as the sum of WDP and SPM, so the literature lacks separate data on the OPP concentrations for only the SPM. Compared to previous studies on OPP occurrence in Italian Rivers, the total OPP concentration found in the Sarno River (Italy) (n.d.—5.16 ng L^−1^) by Montuori et al. [53] were lower than those detected in the Sele River (n.d. to 20.37 ng L^−1^), but comparable to those found in the Tiber River (n.d.—19.98 ng L^−1^) [54] and in the Volturno River (n.d.—19.57 ng L^−1^) [55]. Therefore, compared with the study by Cruzeiro et al., the amount found in the Sele River for chlorpyrifos (mean value = 0.132 mg/kg) was higher than that found in the Tagus River (mean value = 131.32 mg/kg) and also for diazinon (mean value = 2.74 mg/kg), dimethoate (mean value = 0.50 mg/kg), fenitrothion (mean value = 110.23 mg/kg), and malathion (mean value = 1.35 mg/kg) [8].

In addition, since most of the earlier studies were carried out on the water matrix (as the sum of WDP and SPM), the concentrations obtained in water (as the sum of WDP and SPM) were also evaluated. In particular, total OPP concentrations were within the range of 1.02–63.61 ng L^−1^ (mean value 13.55 ± 14.01 ng L^−1^, as sum WDP + SPM). In detail, the concentrations found for the individual analytes were in the range of n.d.—4.81 ng L^−1^ (mean value 1.02 ± 1.39 ng L^−1^) for diazinon, n.d.—14.19 ng L^−1^ (mean value 2.14 ± 2.97 ng L^−1^) for dimethoate, n.d.—4.42 ng L^−1^ (mean value 1.03 ± 1.23 ng L^−1^) for malathion, 0.17–20.65 ng L^−1^ (mean value 5.32 ± 5.22 ng L^−1^) for chlorpyrifos, n.d.—4.57 ng L^−1^ (mean value 0.85 ± 0.90 ng L^−1^) for pirimiphos-methyl, n.d.—7.30 ng L^−1^ (mean value 1.13 ± 1.36 ng L^−1^) for fenitrothion, n.d.—1.84 ng L^−1^ (mean value 0.58 ± 0.53 ng L^−1^) for methidathion, n.d.—6.67 ng L^−1^ (mean value 0.69 ± 1.41 ng L^−1^) for tolclofos-methyl, and n.d.—3.63 ng L^−1^ (mean value 0.79 ± 0.72 ng L^−1^) for parathion.

Compared to previous studies, the concentrations found in the water samples of the Sele River (as a sum of WDP and SPM) were higher than those detected by Charalampous et al. [77] in the Asopos River (Greece) for malathion (range 0.01–0.04 ng L^−1^) and by Aguilar et al. [78], who found lower levels in Jùcar River (Spain) for diazinon (range n.d.—2.00 ng L^−1^) and chlorpyrifos (range 1.95–8.68 ng L^−1^). Moreover, compared with this study, the OPP concentrations detected in the Sarno River were lower for diazinon (range 0.32–2.01 ng L^−1^), dimethoate (range 1.77–6.23 ng L^−1^), tolclofos-methyl (range 3.17–4.20 ng L^−1^), chlorpyrifos (range 1.12–12.16 ng L^−1^), and fenitrothion (range 1.51–4.79 ng L^−1^), but comparable for malathion (range 1.75–5.14 ng L^−1^) and methidation (range 0.50–2.31 ng L^−1^) [53]. In addition, the OPP concentrations detected in this study (as sum of WDP and SPM) were lower than those found for all analysed pesticides in the Tiber River [54], but comparable with those of the Volturno River (range 0.12–65.09 ng L^−1^) [55].

The OPP concentrations obtained for SED samples are indicated in Table 4. The total OPP amount detected ranged from 0.58 ± 0.21 (Station 7, 1500 m westward) to 18.93 ± 0.91 ng g^−1^ dw (Station 1, river mouth), with a mean value of 7.05 ± 6.65 ng g^−1^ dw. Only three of the pesticides analysed were found in the SED samples: chlorpyrifos, tolclofos-methyl, and pirimiphos-methyl. In detail, the concentrations found for the individual analytes were in range of 0.45–14.70 ng g^−1^ dw (mean value 4.74 ± 4.81 ng g^−1^ dw) for chlorpyrifos, n.d.—5.49 ng g^−1^ dw (mean value 1.27 ± 1.65 ng g^−1^ dw) for tolclofos-methyl and 0.13–2.72 ng g^−1^ dw (mean value 1.04 ± 0.84 ng g^−1^ dw) for pirimiphos-methyl. The highest concentrations detected were 11.21 ± 0.72 ng g^−1^ dw for chlorpyrifos, 5.49 ± 0.49 ng g^−1^ dw for tolclofos-methyl, and 1.27 ± 1.65 ng g^−1^ dw for pirimiphos-methyl. The predominance of chlorpyrifos in the sediment samples (67.25% of total OPP concentrations) could be due to a high affinity to soil and sediment components (high K_oc_ value) and its high soil degradation time (DT_50_ = 386 days), as shown in Table 5, which make it very persistent in sediment, although it is no longer used as a pesticide. Compared with previous studies from literature, the concentrations found in the Sele River were lower than those detected for chlorpyrifos in the Zio River (Togo) (range 0.82–26.93 ng g^−1^ dw) by Mawussi et al. [79], in the Berre Lagoon (France) (range 0.39–130.97 ng g^−1^ dw) by Kanzari et al. [80], and in Turia and Jucar Rivers (Spain) (range 4.51–55.95 ng g^−1^ dw) by Ccanccapa et al. [81]. In addition, the concentrations found in the sediment samples of the Sele River were higher than those of the Sarno River in chlorpyrifos (0.19–2.60 ng g^−1^ dw) and tolclofos-methyl (0.89–1.89 ng g^−1^ dw) [53]. Moreover, the concentrations found in the sediment of the Tiber River were higher than those detected in this study for diazinon (range 0.18–0.54 ng g^−1^ dw), chlorpyrifos (range 0.26–32.85 ng g^−1^ dw), pirimiphos-methyl (range 0.18–8.69 ng g^−1^ dw), and tolclofos-methyl (range 0.28–26.37 ng g^−1^ dw) [54]. Finally, the OPP concentrations of the Volturno River were comparable to those of this study for chlorpyrifos (range 0.98–16.27 ng g^−1^ dw), but lower than those of pirimiphos-methyl (range 0.21–3.97 ng g^−1^ dw) and tolclofos-methyl (range 0.21–7.29 ng g^−1^ dw) [55].

### 3.2. OPP Distribution between WDP, SPM, and SED Samples

The ratios of the total concentrations in WDP to those in SPM samples were evaluated. In most sampling sites and seasons the concentration ratios [∑OPPs_WDP_]/[∑OPPs_SPM_] (ng L^−1^) were > 1, suggesting that the total OPP amount in WDP samples was higher than that in SPM samples for most sites. In fact, the total concentration of OPPs detected in WDP (392.59 ng L^−1^, as sum of all sites and seasons) was higher than that found for SPM (149.53 ng L^−1^, as sum of all sites and seasons) samples. These results can certainly be explained considering the OPP’s chemical and physical characteristics (Table 1); just think, for example, of dimethoate, which exhibits a very high solubility in water, a low soil organic carbon partition coefficient, very low persistence in sediment (soil degradation DT_50_ = 2.5 days), and a high tendency to migrate from sediment to a dissolved phase (water-sediment DT_50_ = 15.5 days—moderately fast). In addition, the presence of malathion in WDP can be associated to its relatively high solubility in water (148 mg L^−1^), explained also by its hydrophilic nature (Log*K_ow_* = 2.36) and non-persistence in sediment due to its very low soil degradation (DT_50_ = 0.17 days) (Table 5).

Moreover, the ratios of total concentrations in water to those in SED samples were evaluated.

In most sampling sites and seasons, the concentration ratios [∑OPPs_water_]/[∑OPPs_SED_] were > 1, suggesting that the total amount of OPPs in water samples was higher than that in SED samples for most sites. Therefore, the total concentrations decreased from WDP to SPM, and more to sediment, for most of the analytes considered; that is except for chlorpyrifos, tolclofos-methyl, and pirimiphos-methyl, which are the only ones found in the sediment, and for which the ratios are in the most sites <1, suggesting no fresh inputs of these chemicals in the Sele River estuary, which might be attributed to the resuspension or sedimentation processes that cause the movement of such compounds between the sediment where they can be adsorbed, as well as the aqueous phase. This is also confirmed by a similar concentration pattern found both in the water and sediment.

### 3.3. Loads and Spatial-Temporal Distribution into the Mediterranean Sea

The total load contribution of OPPs to the central Mediterranean Sea from the Sele River mouth was obtained as the product of the total concentrations for the average annual flow and was calculated at about 61.46 kg year^−1^. In detail, the load was 4.77 kg year^−1^ for diazinon, 12.43 kg year^−1^ for dimethoate, 6.46 kg year^−1^ for malathion, 19.01 kg year^−1^ for chlorpyrifos, kg year^−1^ for pirimifos-methyl, 3.40 kg year^−1^ for fenitrothion, 2.16 kg year^−1^ for methidathion, 5.33 kg year^−1^ for tolclofos-methyl, and 3.89 kg year^−1^ for parathion. These results indicate that, even if the concentrations of individual OPPs found were low, the total load of OPPs from the Sele River in the Mediterranean Sea was significant, so this river could represent an important point source of OPP discharge into the sea. Compared with loads evaluated in previous studies, the total load contribution of OPPs from the Sele River was higher than that estimated from the Sarno River (Italy) at about 48.06 kg year^−1^, but lower than that estimated from the Volturno River (Italy) at about 71.81 kg year^−1^ to the Central Mediterranean Sea [54,55].

**Figure 3:**
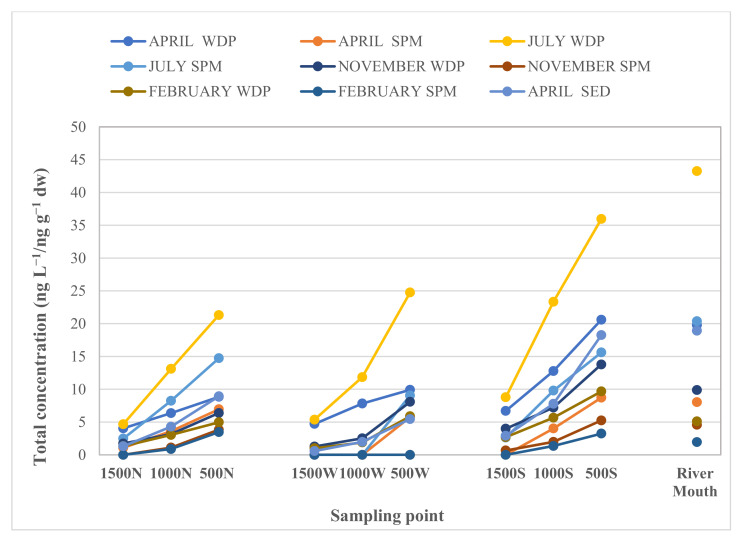
Spatial and temporal concentration of OPPs in the water dissolved phase (WDP, ng L^−1^), suspended particulate matter (SPM, ng L^−1^) and sediments (SED, ng g^−1^ dry wt) of the Sele River, Southern Italy in 10 sampling sites (river mouth; 500N: nord estuary at 500 m; 1000N: nord estuary at 1000 m; 1500N: nord estuary at 1500 m; 500W: west estuary at 500 m; 1000W: west estuary at 1000 m; 1500W: west estuary at 1500 m; 500S: south estuary at 500 m; 1000S: south estuary at 1000 m; and 1500S: south estuary at 1500 m).

The spatial distribution of OPPs from the Sele River was evaluated by comparing the amount of samples collected in 10 sampling sites throughout the period considered (Figure 3).

**Figure 4:**
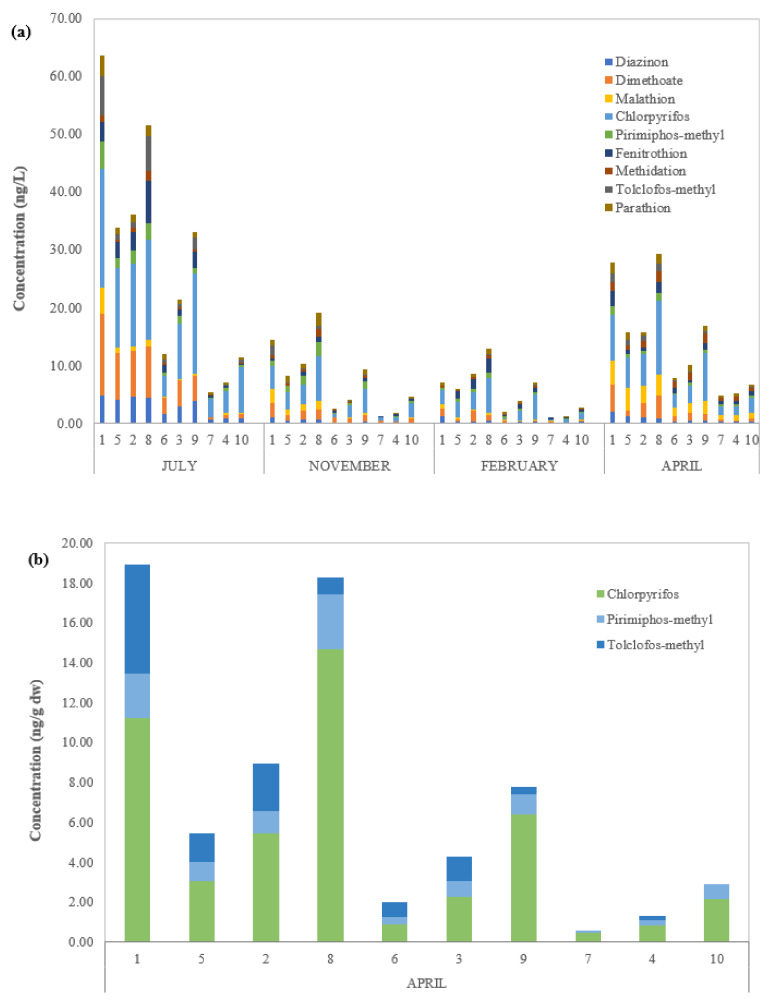
(**a**) Concentrations of OPPs (ng L^−1^) detected in water (WDP+SPM) of the Sele River estuary in four seasons (July, November, February, and April) and (**b**) in sediment (SED) (ng g^−1^ dry wt) in April at 10 sampling sites (1: river mouth; 2: nord estuary at 500 m; 3: nord estuary at 1000 m; 4: nord estuary at 1500 m; 5: west estuary at 500 m; 6: west estuary at 1000 m; 7: west estuary at 1500 m; 8: south estuary at 500 m; 9: south estuary at 1000 m; and 10: south estuary at 1500 m).

Figure 4 shows the concentrations of OPPs detected in the water dissolved phase (WDP, ng L^−1^), suspended particulate matter (SPM, ng L^−1^), and sediments (SED, ng g^−1^ dw) of the Sele River, Southern Italy, in 10 sampling sites. The highest concentrations were found for water at the river mouth (28.25 ng L^−1^ as WDP + SPM mean value of four seasons), then gradually decreased from 27.22 ng L^−1^ (WDP + SPM mean values of four seasons) at 500S (500 m from the estuary to the south) to 17.63 ng L^−1^ (WDP + SPM mean values of four seasons) at 500N (500 m from the estuary to the north). The highest levels were found at the mouth and then decreased moving away from the mouth, with the lowest amount recorded at 1500N and 1500W (1500 m to the north and to the west, respectively) (Figure 4a,b). In fact, Figure 4a,b shows that for both the water matrix and the sediment, the highest total concentrations were detected at Sites 1 (river mouth) and 8 (south estuary at 500 m), in all sampling seasons. Particularly, the OPP loads moved from the Sele River mouth southwards to the Mediterranean Sea.

**Figure 5:**
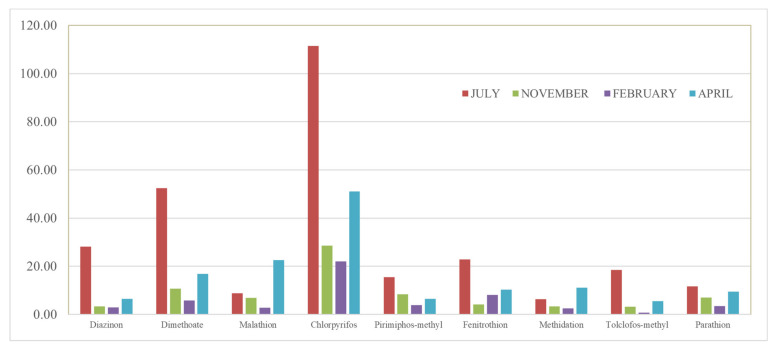
Total OPP concentrations detected in water (sum of WDP and SPM-ng L^−1^) during the four sampling seasons (July, November, February, and April) at 10 sampling sites.

The temporal variation was evaluated by comparing the amount of samples collected at the sampling sites throughout the four sampling seasons, without rain (July and April) and with rain (November and February). For water, the total OPP concentrations were higher in the warm seasons (July and April) than in cold seasons (November and February) (Figure 4a). In fact, as shown in Figure 5, for water samples, the mean total pesticide concentrations detected in WDP + SPM (for all sites) decreased in the following order: 27.52 ng L^−1^ in July, 13.98 ng L^−1^ in April, 7.52 ng L^−1^ in November, and 5.19 ng L^−1^ in February. In July, high total of OPP concentrations observed (275.24 ng L^−1^ in WDP + SPM as sum of total OPP concentrations detected in all sampling sites) could be due to the more intensive agricultural activities typical of the warmer period, along with the lower flow conditions due to low rainfall. In fact, summer is a critical season, for the control of crop diseases and the consumption of pesticides is huge, which results in a higher concentration in summer than in winter [34]. However, the minimum pesticide application resulted in lower total concentrations recorded in February (51.91 ng L^−1^ as sum of WDP and SPM), which also coincided, along with December, with the rainiest period. In fact, the high flow condition in winter could result in dilution of the pesticide concentrations in surface water. According to the above study, Xu et al. and Wang et al. noted higher levels of pesticides in warm seasons and associated these results to the intense agricultural practices and the lower frequency of the precipitation [30,34]. In addition, compared to previous studies, the temporal trends of OPPs observed in the Sele River estuary were similar to those noticed in the Sarno and Volturno Rivers [54,55].

**Figure 6:**
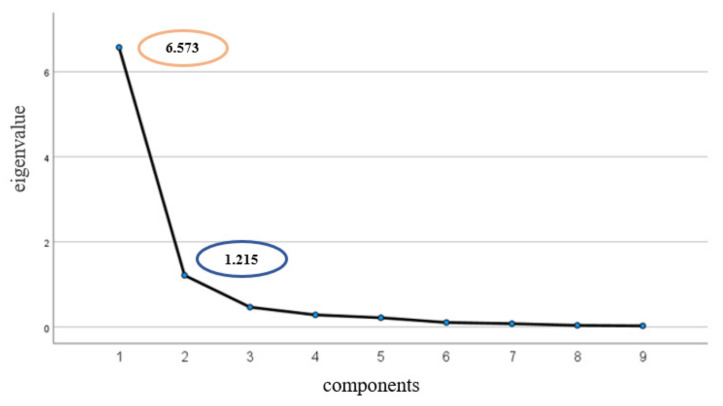
Principal component analysis: scree plot of the data collected from the Sele River and its estuary.

**Figure 7:**
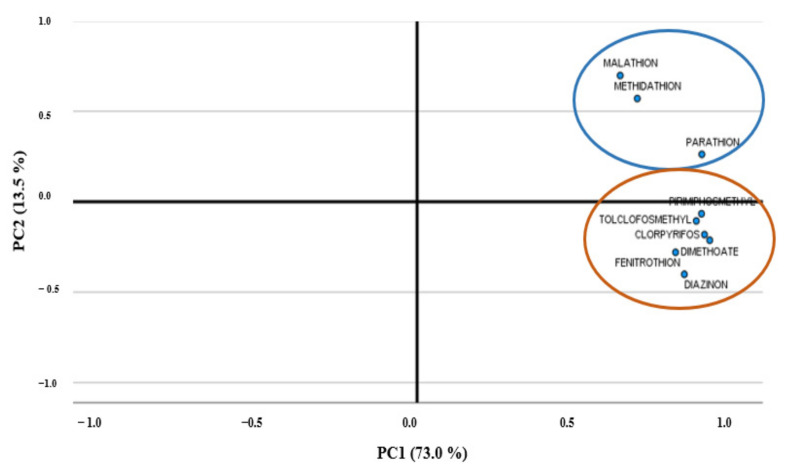
Principal component analysis: loading plot for the first and second principal components (PCs).

For quantitative assessment, the PCA was employed for the OPPs evaluated in water samples, considering their concentrations as the sum of WDP and SPM. According to the eigenvalues and the cumulative proportions of the explained variance obtained, the first two PCs, explaining up to 86.5% of the total variability, were retained. In particular, the eigenvalues found were 6573 and 1215, and the cumulative proportions were 73.0 and 13.5%, respectively, for the two PCs (Figure 6). Moreover, Figure 7 shows the loading plot for the first and second PCs. Particularly, it suggests that the first component could be explained as the detected OPP concentrations, while the second component was associated with the malathion and methidathion. In fact, for the first component, all positive loadings were obtained, therefore the PC could be interpreted as the weighted average of the variables, and it was therefore associated to the assessment of the total pollution level.

On the other hand, the second component focused attention on the contrast between negative coefficients associated to the pesticides still on the market, or recently withdrawn (chlorpyrifos), and positive coefficients related to the pesticides no longer in use. In particular, negative coefficients are obtained for all OPPs except for malathion, methidathion, and parathion, for which positive coefficients were found. In addition, Figure 7 suggests a substantial independence between the pesticides on the market and those no longer in use.

### 3.4. Risk Assessment of OPPs in the Sele River and Estuary

Concerns for the environmental and human health effects of OPPs encouraged many countries to implement environmental quality standards (EQS) for priority substances in surface waters. Moreover, regional regulatory actions may be less appropriate in other regions because the contamination levels and distribution of pollutants vary based on seasonal variation and different patterns of use. Consequently, countries or regions should have their own legislative guidelines and standards to regulate environmental pesticide pollution. The OPP concentrations observed in the water (as a sum of WDP and SPM) of the Sele River were below the criterion maximum concentration (CMC), established under the United States Environmental Protection Agency for chlorpyrifos, malathion, and diazinon (0.083, 0.100 and 0.170 µg L^−1^, respectively) [82]. In fact, the maximum concentrations obtained for these pesticides were 0.021 µg L^−1^, 0.004 µg L^−1^, and 0.005 µg L^−1^, respectively.

**Figure 8:**
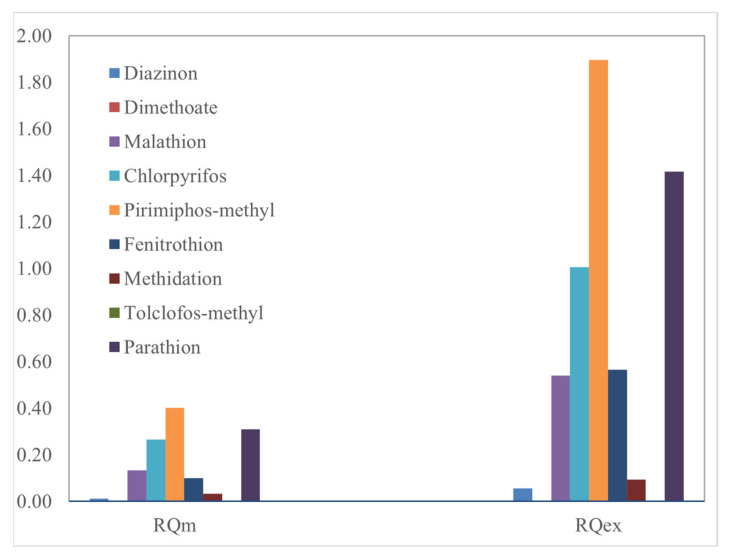
Risk quotients (*RQ_m_* and *RQ_ex_*) of the detected OPPs in the water (WDP+SPM) of the Sele River estuary, Southern Italy.

The *RQ* method was used to evaluate the possible adverse effect of OPP contamination in the Sele River ecosystem. The method was determined by applying NOEC and *EC*_50_ (or *LC*_50_) values for different trophic levels. The results obtained for the detected chemicals are indicated in Table 6 and illustrated in Figure 8. None of the OPPs studied posed high risk (*RQ_m_* > 1) for the general-case scenario. Particularly, using mean MECs, negligible risk (*RQ_m_* < 0.01) was revealed for dimethoate and tolclofos-methyl (*RQ_m_* = 0.0004), while for diazinon (*RQ_m_* = 0.0132), fenitrothion (*RQ_m_* = 0.0995), and methidation (*RQ_m_* = 0.0340), a low risk (0.01 < *RQ_m_* <0.1) was assessed. Moreover, medium risk was related to chlorpyrifos (*RQ_m_* = 0.2668), malathion (*RQ_m_* = 0.1332), parathion (*RQ_m_* = 0.3095), and pirimiphos-methyl (*RQ_m_* = 0.4020). For the worst-case scenario, the *RQ_ex_* using the maximum concentrations detected was calculated. As shown in Table 6, using maximum MECs, chlorpyrifos, pirimiphos-methyl, and parathion exhibited *RQ_ex_* >1, indicating that a potential high risk could be associated with chronic exposure to these chemicals in the Sele River ecosystem. Medium risk was associated with fenitrothion (*RQ_ex_* = 0.5658) and malathion (*RQ_ex_* = 0.5407), unlike diazinon and methidathion for which 0.01 < *RQ_ex_* < 0.1 (low risk) was found. No risk for dimethoate and tolclofos-methyl was assessed (*RQ_ex_* < 0.01). In addition, the risk quotient of the OPP mixture (*RQ_mix_*), evaluated as the sum of the individual *RQ_m_* values of each OPP, was 12590, indicating a potential high risk.

The results show that, although high-risk indices for the general-case scenario were not observed, alarm indices were observed in the worst-case scenario, thus considering the maximum concentrations obtained for individual analytes. In this case, in fact, values of *RQ_ex_* greater than 1 were found for three of the analytes studied, namely chlorpyrifos, pyrimifos-methyl, and parathion. The results obtained could be explained by loads, in fact, the major contribution of OPPs was that of chlorpyrifos (30.92% of the total OPP load), which despite being banned, continues to persist both in the sediment and in water phase because of the resuspension phenomena and changes in flow during the various seasons. However, the results obtained for the Sele River, with regard to chlorpyrifos, were found to be much lower than those obtained under chronic exposure (*RQ_m_* = 1.9643 and *RQ_ex_* = 11.5643, indicating high risk both in general and worst-case scenarios) by Zainuddin et al., which estimated the ecological risk due to the presence of OPPs in the riverine ecosystem of the Linggi River (Malaysia), where this pesticide is still widely used for crops [16]. In addition, Wee et al. also deduced that the chlorpyrifos posed a potential risk (*RQ_m_* = 1.44; *RQ_ex_* = 4.83) for aquatic organisms in the Langat River (Malaysia) [19]. Compared with previous studies from Italy, in the Tiber River, no OPPs posed high risk (*RQ_m_* < 1), but using maximum MECs, higher *RQ_ex_* values (potential high risk) compared to those of this study were assessed for malathion (*RQ_ex_* = 5.3781), pirimiphos-methyl (*RQ_ex_* = 5.1688), chlorpyrifos (*RQ_ex_* = 1.5593), and fenitrothion (*RQ_ex_* = 1.4726) [54]. Moreover, also in the Volturno River, no OPPs presented an RQ_m_ higher than 1, but using maximum MECs, the *RQ_ex_* was higher than that of the Sele River for pirimiphos-methyl (*RQ_ex_* = 2.3952) and similar for chlorpyrifos (*RQ_ex_* = 1.0984) [55].

In view of the above, it is clear that the ecological risk of OPPs in the river ecosystem varies widely globally and certainly depends on pesticide loading sources, as well as pesticide degradation rates and meteorological events. Additionally, the source of pesticide pollution should be identified and the pesticide discharge should be controlled. The *RQ* results show that the potential risk from OPPs should not be neglected, even though they are in compliance with regulations. Overall, risk assessment for OPPs is important as part of the preliminary efforts to preserve and protect the aquatic ecosystem and human health. Risk assessment will help competent authority to improve decision making and policy implementation. Therefore, investigation on the risk assessment of mixed pesticides for aquatic species and human health is constantly required.

## 4. Conclusions

The intensive use of organophosphorus pesticides is a reason of concern in many countries as a potential threat to environmental sustainability, mainly due to the chemical characteristics of inertia and posing time of some substances. Consequently, monitoring programmes are needed to document the hazards in order to evaluate their impact and preserve the aquatic ecosystem. This study is the first to offer helpful information about the amount of OPP contamination in the Sele River estuary. The results indicate that higher levels were found in water (as sum of WDP + SPM) than in sediment samples due to their physio-chemical properties, but also due to resuspension phenomena that occur as a result of changes in flow during different seasons. Therefore, the water/sediment ratios were in most cases >1, indicating higher OPPs in water than in sediment. Only for chlorpyrifos, tolclofos-methyl, and pirimiphos-methyl, the only ones found in the sediment samples, the ratios were <1, suggesting no fresh inputs of these chemicals in the Sele River estuary. In addition, the results obtained about the spatial and seasonal distribution show higher levels of OPP at the river mouth and at 500 m southward of the river mouth in the summer period because of the increased use of pesticides and low flow conditions due to rainfall deficit. Regarding the risk assessment, despite the compliance with regulations, it was revealed that chlorpyrifos, pirimifos-methyl, and parathion could pose a threat to the aquatic life. Taking this into consideration, the sources of pesticides and the potential risks posed by mixed pesticides to aquatic ecosystems and human health require further investigation. The results of this study can provide some guidance for water resources managers and regulatory issues, such as monitoring, control, and management of organophosphorus pesticides in the future.

## Figures and Tables

**Table 1:** Molecular formula and weight, chemical structure, octanol–water partitioning coefficient (LogK_ow_), solubility in water at 20 °C (mg L^−1^) and uses of the detected pesticides.

Pesticide Name	Molecular Formula	Molecular Weight	Chemical Structure	LogK*_ow_*	Solubility in Water at 20 °C (mg L^−1^)	Uses
Parathion	C_10_H_14_NO_5_PS	291.26	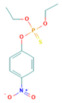	3.83 ^a^	12.4 ^c^	Insecticide Acaricide Avicide
Malathion	C_10_H_19_O_6_PS_2_	330.40	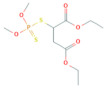	2.36 ^a^	148 ^c^	Insecticide
Chlorpyrifos	C_9_H_11_Cl_3_NO_3_PS	350.60	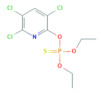	4.96 ^a,b^	1.05 ^c^	Insecticide Acaricide
Diazinon	C_12_H_21_N_2_O_3_PS	304.35	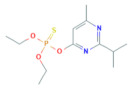	3.81 ^a^	60 ^c^	Acaricide Nematicide
Fenitrothion	C_9_H_12_NO_5_PS	277.24	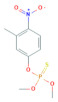	3.30 ^a^	19 ^c^	Insecticide Acaricide
Methidathion	C_6_H_11_N_2_O_4_PS_3_	302.30	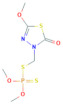	2.20 ^b^	240 ^c^	Insecticide Acaricide
Pirimiphos-methyl	C_11_H_20_N_3_O_3_PS	305.34	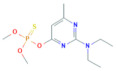	4.12 ^a^	11 ^c^	Insecticide Acaricide
Tolclofos-methyl	C_9_H_11_Cl_2_O_3_PS	301.10	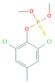	4.56 ^a^	0.71 ^c^	Fungicide
Dimethoate	C_5_H_12_NO_3_PS_2_	229.3	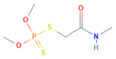	0.78 ^a^	25,900 ^c^	Insecticide Acaricide

Molecular Weight: g mol^−1^. *K_ow_*: octanol–water partitioning coefficient. ^a^ Hazardous Substances Data Bank (HSDB) [60]. ^b^ ILO International Chemical Safety Cards (ICSC) [61]. ^c^ Pesticide Properties DataBase (PPDB), 2021 [62].

**Table 2:** Description of the sampling sites and OPP concentration range (ng L^−1^) detected in the water dissolved phase (WDP) of the Sele River, Southern Italy, during four sampling seasons.

Sampling Location			Organophosphate Pesticide Concentration Range (ng L^−1^)									
Site Number Identification	Site Characteristics	Site Location	Diazinon	Dimethoate	Malathion	Clorpyrifos	Pirimiphos-Methyl	Fenitrothion	Methidathion	Tolclofos-Methyl	Parathion	Total
1 (river water)	Sele River mouth	40°28′55″ N 14°56′33″ E	0.64–3.17	1.01–10.08	0.53–3.24	1.61–14.08	0.38–3.03	n.d.–2.19	0.28–0.83	0.14–4.03	0.38–2.83	5.10–43.24
2 (sea water)	River mouth 500 m north	40°29′04″ N 14°56′14″ E	0.26–2.84	0.38–5.04	0.21–2.26	1.65–8.79	0.31–1.00	0.52–1.47	0.22–0.39	0.14–0.45	0.29–1.01	4.96–21.32
3 (sea water)	River mouth 1000 m north	40°29′12″ N 14°55′56″ E	n.d.–1.50	0.13–2.41	0.14–0.96	1.14–6.75	0.21–0.51	0.20–0.55	n.d–0.90	n.d.–0.31	0.31–0.71	3.04–13.12
4 (sea water)	River mouth 1500 m north	40°29′20″ N 14°55′38″ E	n.d–0.42	n.d–0.37	n.d–0.69	0.41–2.21	0.20–0.42	0.21–0.56	n.d.–0.44	n.d.–0.17	0.20–0.42	1.32–4.68
5 (sea water)	River mouth 500 m west	40°28′55″ N 14°56′12″ E	0.30–2.97	0.38–5.85	0.25–2.69	2.62–9.59	0.41–1.20	n.d.–1.96	0.25–0.53	n.d.–0.65	0.20–1.20	5.90–24.76
6 (sea water)	River mouth 1000 m west	40°28′55″ N 14°55′50″ E	0.13–1.57	0.23–2.78	n.d.–1.37	0.17–3.41	n.d.–0.62	0.25–1.27	n.d.–1.03	n.d.–0.59	0.17–0.82	1.88–11.85
7 (sea water)	River mouth 1500 m west	40°28′55″ N 14°55′28″ E	0.12–0.53	0.10–0.42	n.d.–0.78	0.32–3.21	n.d.–0.26	0.27–0.65	n.d.–0.45	n.d.–0.16	n.d.–0.26	1.02–5.37
8 (sea water)	River mouth 500 m south	40°28′47″ N 14°56′16″ E	0.37–3.05	0.95–6.02	0.52–2.40	3.56–11.78	0.92–1.95	0.73–4.92	0.52–1.21	0.20–3.89	0.92–1.95	9.67–35.96
9 (sea water)	River mouth 1000 m south	40°28′39″ N 14°55′56″ E	0.16–2.61	0.29–3.24	0.22–1.77	2.88–11.33	0.34–0.99	0.57–1.86	0.36–1.17	0.12–1.42	0.43–0.99	5.67–23.35
10 (sea water)	River mouth 1500 m south	40°28′30″ N 14°55′38″ E	0.14–0.80	0.29–0.77	0.11–0.89	1.11–5.30	0.19–0.41	0.27–0.82	n.d.–0.61	n.d.–0.58	0.22–0.41	2.71–8.78

n.d.: not detected.

**Table 3:** Description of the sampling sites and OPP concentration range (ng L^−1^) detected in the suspended particulate matter (SPM) samples from the Sele River, Southern Italy during four sampling seasons.

Sampling Location			Organophosphate Pesticides Concentration Ranges (ng L^−1^)									
Site Number Identification	Site Characteristics	Site Location	Diazinon	Dimethoate	Malathion	Clorpyrifos	Pirimiphos-Methyl	Fenitrothion	Methidathion	Tolclofos-Methyl	Parathion	Total
1 (river water)	Sele River mouth	40°28′55″ N 14°56′33″ E	0.33–1.63	0.43–4.11	0.26–1.40	0.81–6.58	n.d.—1.54	n.d.—1.23	n.d.—0.46	n.d.—2.64	0.14–0.92	1.96–20.37
2 (sea water)	River mouth 500 m north	40°29′04″ N 14°56′14″ E	n.d.—1.75	0.85–2.74	n.d.—0.67	1.27–5.47	n.d.—1.24	n.d.—1.76	n.d.—0.72	n.d.—0.43	n.d.—0.46	3.48–14.63
3 (sea water)	River mouth 1000 m north	40°29′12″ N 14°55′56″ E	n.d.—1.33	n.d.—2.08	n.d.—0.83	0.60–3.01	n.d.—0.78	n.d.—0.60	n.d.—0.36	n.d.—0.21	n.d.—0.74	0.89–8.25
4 (sea water)	River mouth 1500 m north	40°29′20″ N 14°55′38″ E	n.d.—0.34	n.d.—0.30	n.d.—0.23	n.d.—1.59	n.d.—0.25	n.d.	n.d.—0.23	n.d.	n.d.—0.13	n.d.—2.48
5 (sea water)	River mouth 500 m west	40°28′55″ N 14°56′12″ E	n.d.—1.06	n.d.—2.26	n.d.—1.17	n.d.—4.14	n.d.—0.49	n.d.—0.83	n.d.—0.26	n.d.—0.30	n.d.—0.97	n.d.—9.06
6 (sea water)	River mouth 1000 m west	40°28′55″ N 14°55′50″ E	n.d.	n.d.	n.d.	n.d.	n.d.	n.d.	n.d.	n.d.	n.d.	n.d.
7 (sea water)	River mouth 1500 m west	40°28′55″ N 14°55′28″ E	n.d.	n.d.	n.d.	n.d.	n.d.	n.d.	n.d.	n.d.	n.d.	n.d.
8 (sea water)	River mouth 500 m south	40°28′47″ N 14°56′16″ E	n.d.—1.32	n.d.—2.81	n.d.—1.08	2.44–5.57	n.d.—0.78	n.d.—2.38	n.d.—0.63	n.d.—2.18	n.d.—0.88	3.24–15.62
9 (sea water)	River mouth 1000 m south	40°28′39″ N 14°55′56″ E	n.d.—1.26	n.d.—0.84	n.d.—0.50	1.20–5.98	n.d.—0.40	n.d.—0.93	n.d.—0.62	n.d.—0.62	n.d.- 0.40	1.36–9.78
10 (sea water)	River mouth 1500 m south	40°28′30″ N 14°55′38″ E	n.d	n.d.	n.d.	n.d.—2.64	n.d.	n.d.	n.d.	n.d.	n.d.	n.d.—2.64

n.d.: not detected.

**Table 4:** Description of sampling sites and OPP concentration with standard deviations (SD) detected in the sediment samples (SED) (ng g^−1^ dw) of Sele River, southern Italy.

Sampling Location			Sampling Season	Organophosphate Pesticides Concentration (ng g^−1^ dw) ± Standard Deviations (SD)									
Site Number Identification	Site Characteristics	Site Location	Campaigns	Diazinon	Dimethoate	Malathion	Clorpyrifos	Pirimiphos -Methyl	Fenitrothion	Methidathion	Tolclofos-Methyl	Parathion	Total
1 (river water)	Sele River mouth	40°28′55″ N 14°56′33″ E	April	n.d.	n.d.	n.d.	11.21 ± 0.72	2.23 ± 0.27	n.d.	n.d.	5.49 ± 0.49	n.d.	18.93 ± 0.91
2 (sea water)	River mouth 500 m north	40°29′04″ N 14°56′14″ E	April	n.d.	n.d.	n.d.	5.43 ± 0.47	1.11 ± 0.14	n.d.	n.d.	2.38 ± 0.21	n.d.	8.93 ± 0.46
3 (sea water)	River mouth 1000 m north	40°29′12″ N 14°55′56″ E	April	n.d.	n.d.	n.d.	2.25 ± 0.33	0.83 ± 0.19	n.d.	n.d.	1.22 ± 0.21	n.d.	4.30 ± 0.30
4 (sea water)	River mouth 1500 m north	40°29′20″ N 14°55′38″ E	April	n.d.	n.d.	n.d.	0.85 ± 0.18	0.26 ± 0.08	n.d.	n.d.	0.21 ± 0.08	n.d.	1.32 ± 0.07
5 (sea water)	River mouth 500 m west	40°28′55″ N 14°56′12″ E	April	n.d.	n.d.	n.d.	3.04 ± 0.20	0.97 ± 0.12	n.d.	n.d.	1.45 ± 0.21	n.d.	5.46 ± 0.53
6 (sea water)	River mouth 1000 m west	40°28′55″ N 14°55′50″ E	April	n.d.	n.d.	n.d.	0.90 ± 0.21	0.35 ± 0.11	n.d.	n.d.	0.74 ± 0.14	n.d.	1.99 ± 0.20
7 (sea water)	River mouth 1500 m west	40°28′55″ N 14°55′28″ E	April	n.d.	n.d.	n.d.	0.45 ± 0.14	0.13 ± 0.07	n.d.	n.d.	nd	n.d.	0.58 ± 0.21
8 (sea water)	River mouth 500 m south	40°28′47″ N 14°56′16″ E	April	n.d.	n.d.	n.d.	14.70 ± 3.18	2.72 ± 0.41	n.d.	n.d.	0.83 ± 0.19	n.d.	18.26 ± 3.76
9 (sea water)	River mouth 1000 m south	40°28′39″ N 14°55′56″ E	April	n.d.	n.d.	n.d.	6.38 ± 0.41	1.01 ± 0.19	n.d.	n.d.	0.39 ± 0.13	n.d.	7.79 ± 0.09
10 (sea water)	River mouth 1500 m south	40°28′30″ N 14°55′38″ E	April	n.d.	n.d.	n.d.	2.16 ± 0.18	0.74 ± 0.18	n.d.	n.d.	n.d.	n.d.	2.91 ± 0.36

n.d.: not detected.

**Table 5:** Soil organic carbon partition coefficient (mL g^−1^) (K_oc_), soil degradation DT_50_ (days), water-sediment DT₅₀ (days), water phase only DT₅₀ (days) of the detected pesticides.

Pesticide Name	K_oc_^a^	Soil Degradation DT_50_ (Days) ^b^		Water-Sediment DT₅₀ (Days) ^b^		Water Phase Only DT₅₀ (Days) ^b^	
Parathion	1580	49	moderately persistent	4.3	fast	3.5	fast
Malathion	229	0.17	non-persistent	0.4	fast	0.4	fast
Chlorpyrifos	5010	386	very persistent	36.5	moderately fast	5	moderately fast
Diazinon	562	9.1	non-persistent	10.4	moderately fast	4.3	fast
Fenitrothion	427	2.7	non-persistent	1.57	fast	1.1	fast
Methidathion	33.9	10	non-persistent	70	moderately fast	6	moderately fast
Pirimiphos-methyl	1000	39	moderately persistent	4.73 ^a^	fast	4.73 ^a^	fast
Tolclofos-methyl	761–1540 ^c^	7.6	non-persistent	15	moderately fast	1.25	fast
Dimethoate	15.8	2.5	non-persistent	15.5	moderately fast	12.6	moderately fast

K_oc_: Soil organic carbon partition coefficient (mL g^−1^). ^a^ U.S. EPA Distributed Structure-Searchable Toxicity (DSSTox) [74]. https://comptox.epa.gov/dashboard/DTXSID4037580 (accessed on 8 March 2022). ^b^ Pesticide Properties DataBase (PPDB), 2021 [62]. http://sitem.herts.ac.uk/aeru/ppdb/en/index.htm (accessed on 12 January 2022). ^c^ PubChem. https://pubchem.ncbi.nlm.nih.gov/compound/91664 (accessed on 7 January 2022).

**Table 6:** Ecotoxicology risk assessment data for the OPPs detected in the Sele River WDP with ecotoxicity endpoints for three trophic levels (algae, aquatic invertebrates, and fish), *PNEC* values (µg/L), and risk quotients (*RQ_m_*, *RQ_ex_*).

Compound	Ecotoxicology Endpoints Trophic Levels (µg/L)			Critical Concentration (µg/L)	AF	*PNEC*	*RQ_m_*	*RQ_ex_*
	*Algae*	*Aquatic Invertebrates*	*Fish*					
Diazinon	>10,000	0.56	700	0.56	10	0.0560	0.0132	0.0567
Dimethoate	32,000	40	400	40	10	4.0000	0.0004	0.0025
Malathion	13,000 (*EC_50_*)	0.06	91	0.06	10	0.0060	0.1332	0.5407
Chlorpyrifos	43	4.6	0.14	0.14	10	0.0140	0.2668	1.0056
Pirimiphos-methyl	1000 (*EC_50_*)	0.08	23	0.08	50	0.0016	0.4020	1.8952
Fenitrothion	100	0.087	88	0.087	10	0.0087	0.0995	0.5658
Methidathion	>200	0.64	10 (*LC_50_*)	0.64	50	0.0128	0.0340	0.0948
Tolclofos-methyl	32	26	12	12	10	1.2000	0.0004	0.0034
Parathion	10,000 (*EC_50_*)	0.1	>98	0.1	50	0.0020	0.3095	1.4161

AF: assessment factor; *PNEC*: predicted no effect concentration (µg/L); *RQ_m_*: risk quotient based on mean concentrations; *RQ_ex_*: risk quotient based on maximum concentrations; data sources: Pesticide Properties DataBase (PPDB), 2021 [62].

## Data Availability

Not applicable.

## Supplementary Materials

The supporting information can be downloaded at: https://www.mdpi.com/article/10.3390/toxics10070377/s1. Table S1: Description of the sampling sites and OPPs concentration (ng L^−^^1^) with standard deviations (SD) detected in the water dissolved phase (WDP) of the Sele River, southern Italy; Table S2: Description of the sampling sites and OPPs concentration (ng L^−^^1^) with standard deviations (SD) detected in the suspended particulate matter (SPM) samples from the Sele River, southern Italy.
