# Supplementary material for: Occurrence, Distribution, and Risk Assessment of Organophosphorus Pesticides in the Aquatic Environment of the Sele River Estuary, Southern Italy
Source: Toxics. 2022 Jul 7;10(7):377. doi: 10.3390/toxics10070377 (PMC9322807; doi:10.3390/toxics10070377)
Supplement: Supplementary file 1 [file toxics-10-00377-s001.zip › toxics-1789413-supplementary.pdf]

SUPPLEMENTARY MATERIAL

Occurrence, distribution and risk assessment of organophosphorus pesticides in the aquatic environment of the Sele River estuary, southern Italy

Paolo Montuori[a]*, Elvira De Rosa[a], Fabiana Di Duca[a], Bruna De Simone[a], Stefano Scippa[a], Immacolata Russo[a], Michele Sorrentino[a], Pasquale Sarnacchiaro[b], Maria Triassi[a]

[a]Department of Public Health, University “Federico II”, Via Sergio Pansini nº 5, 80131 Naples, Italy
[b]Department of Law and Economics, University “Federico II”, Via Cinthia n° 26, 80126 Naples, Italy

*Corresponding author: Department of Public Health, University “Federico II”, Via Sergio Pansini nº 5, 80131 Naples, Italy; email: pmontuor@unina.it

Table S1. Description of the sampling sites and OPPs concentration (ng L^{-1}) with standard deviations (SD) detected in the water dissolved phase (WDP) of the Sele River, southern Italy.

Sampling location			Sampling season	Organophosphate pesticides concentrations (ng L^{-1}) ± standard deviations (SD)									
Site number identification	Site characteristics	Site location	Campaigns	Diazinon	Dimethoate	Malathion	Clorpyrifos	Pirimiphos -Methyl	Fenitrothion	Methidathion	Tolclofos-Methyl	Parathion	Total
1 (river water)	Sele River mouth	40°28′55”N 14°56′33″E	Jul	3,17 ± 0,20	10,08 ± 0,68	3,01 ± 0,17	14,08 ± 0,73	3,03 ± 0,32	2,19 ± 0,24	0,83 ± 0,23	4,03 ± 0,33	2,83 ± 0,41	43,24 ± 1,06
			Nov	0,64 ± 0,13	1,69 ± 0,17	1,77 ± 0,27	2,61 ± 0,15	0,63 ± 0,14	0,34 ± 0,16	0,50 ± 0,13	1,09 ± 0,18	0,63 ± 0,11	9,92 ± 0,41
			Feb	0,76 ± 0,09	1,01 ± 0,16	0,53 ± 0,08	1,61 ± 0,11	0,38 ± 0,08	n.d.	0,28 ± 0,03	0,14 ± 0,04	0,38 ± 0,10	5,10 ± 0,38
			Apr	1,22 ± 0,11	3,26 ± 0,23	3,24 ± 0,11	5,91 ± 0,23	1,02 ± 0,12	2,00 ± 0,17	1,20 ± 0,15	0,90 ± 0,12	1,02 ± 0,22	19,76 ± 0,17
2 (sea water)	River mouth 500m north	40°29′04”N 14°56′14″E	Jul	2,84 ± 0,39	5,04 ± 0,74	0,49 ± 0,19	8,79 ± 0,74	1,00 ± 0,20	1,47 ± 0,35	0,22 ± 0,09	0,45 ± 0,10	1,01 ± 0,20	21,32 ± 0,93
			Nov	0,43 ± 0,06	0,64 ± 0,12	0,63 ± 0,14	2,10 ± 0,23	1,00 ± 0,14	0,52 ± 0,11	0,39 ± 0,06	0,28 ± 0,07	0,40 ± 0,05	6,39 ± 0,49
			Feb	0,26 ± 0,04	0,38 ± 0,07	0,21 ± 0,09	1,65 ± 0,13	0,65 ± 0,10	1,01 ± 0,17	0,28 ± 0,06	0,14 ± 0,05	0,39 ± 0,08	4,96 ± 0,30
			Apr	0,59 ± 0,20	0,56 ± 0,11	2,26 ± 0,13	3,51 ± 0,17	0,31 ± 0,10	0,53 ± 0,10	0,36 ± 0,09	0,43 ± 0,09	0,29 ± 0,09	8,83 ± 0,24
3 (sea water)	River mouth 1000m north	40°29′12”N 14°55′56″E	Jul	1,50 ± 0,26	2,41 ± 0,40	0,21 ± 0,05	6,75 ± 0,38	0,51 ± 0,08	0,43 ± 0,12	0,28 ± 0,08	0,31 ± 0,07	0,71 ± 0,15	13,12 ± 0,70
			Nov	n.d.	0,46 ± 0,11	0,27 ± 0,10	1,26 ± 0,27	0,31 ± 0,07	0,20 ± 0,03	n.d.	0,15 ± 0,06	0,31 ± 0,08	3,17 ± 0,28
			Feb	0,19 ± 0,04	0,13 ± 0,07	0,14 ± 0,06	1,14 ± 0,10	0,21 ± 0,06	0,55 ± 0,12	0,24 ± 0,07	n.d.	0,34 ± 0,07	3,04 ± 0,09
			Apr	0,34 ± 0,06	0,81 ± 0,13	0,96 ± 0,18	1,92 ± 0,21	0,45 ± 0,12	0,46 ± 0,08	0,90 ± 0,20	0,19 ± 0,05	0,35 ± 0,10	6,38 ± 0,66
4 (sea water)	River mouth 1500m north	40°29′20”N 14°55′38″E	Jul	0,42 ± 0,08	0,37 ± 0,09	0,26 ± 0,06	2,21 ± 0,25	0,42 ± 0,06	0,37 ± 0,07	0,07 ± 0,02	0,13 ± 0,05	0,42 ± 0,02	4,68 ± 0,13
			Nov	n.d.	0,30 ± 0,12	n.d.	0,67 ± 0,14	0,20 ± 0,07	0,30 ± 0,09	n.d.	n.d.	0,20 ± 0,09	1,74 ± 0,13
			Feb	n.d.	n.d.	0,12 ± 0,05	0,41 ± 0,08	0,21 ± 0,06	0,21 ± 0,04	n.d.	n.d.	0,21 ± 0,08	1,32 ± 0,21
			Apr	0,20 ± 0,09	0,27 ± 0,09	0,69 ± 0,14	1,05 ± 0,17	0,33 ± 0,10	0,56 ± 0,14	0,44 ± 0,12	0,17 ± 0,06	0,33 ± 0,08	4,04 ± 0,06
5 (sea water)	River mouth 500m west	40°28′55”N 14°56′12″E	Jul	2,97 ± 0,40	5,85 ± 0,78	0,92 ± 0,32	9,59 ± 0,95	1,20 ± 0,46	1,96 ± 0,36	0,41 ± 0,15	0,65 ± 0,29	1,20 ± 0,19	24,76 ± 1,78
			Nov	0,46 ± 0,13	0,97 ± 0,15	0,95 ± 0,16	3,04 ± 0,09	1,08 ± 0,20	n.d.	0,26 ± 0,08	0,32 ± 0,07	1,00 ± 0,21	8,09 ± 0,44
			Feb	0,30 ± 0,10	0,38 ± 0,08	0,25 ± 0,05	2,62 ± 0,16	0,60 ± 0,14	1,29 ± 0,21	0,25 ± 0,10	n.d.	0,20 ± 0,07	5,90 ± 0,59
			Apr	0,68 ± 0,18	0,66 ± 0,15	2,69 ± 0,18	3,27 ± 0,12	0,41 ± 0,12	0,67 ± 0,15	0,53 ± 0,15	0,59 ± 0,14	0,41 ± 0,09	9,91 ± 0,11
6 (sea water)	River mouth 1000m west	40°28′55”N 14°55′50″E	Jul	1,57 ± 0,28	2,78 ± 0,28	0,31 ± 0,09	3,41 ± 0,34	0,62 ± 0,17	1,27 ± 0,12	0,48 ± 0,13	0,59 ± 0,13	0,82 ± 0,20	11,85 ± 0,23
			Nov	0,14 ± 0,05	0,76 ± 0,13	n.d.	0,75 ± 0,10	n.d.	0,30 ± 0,08	n.d.	n.d.	0,17 ± 0,05	2,54 ± 0,23
			Feb	0,13 ± 0,05	0,23 ± 0,06	0,24 ± 0,08	0,17 ± 0,06	0,41 ± 0,08	0,25 ± 0,06	0,21 ± 0,06	n.d.	0,23 ± 0,06	1,88 ± 0,16
			Apr	0,19 ± 0,06	1,07 ± 0,14	1,37 ± 0,21	2,27 ± 0,21	0,26 ± 0,12	0,97 ± 0,16	1,03 ± 0,10	0,21 ± 0,06	0,46 ± 0,11	7,84 ± 0,58
7 (sea water)	River mouth 1500m west	40°28′55”N 14°55′28″E	Jul	0,53 ± 0,11	0,42 ± 0,06	n.d.	3,21 ± 0,24	0,26 ± 0,08	0,34 ± 0,08	0,28 ± 0,05	n.d.	0,26 ± 0,06	5,37 ± 0,21
			Nov	0,12 ± 0,06	0,34 ± 0,08	n.d.	0,53 ± 0,12	n.d.	0,27 ± 0,07	n.d.	n.d.	n.d.	1,28 ± 0,15
			Feb	0,13 ± 0,06	0,10 ± 0,03	0,14 ± 0,07	0,32 ± 0,10	n.d.	0,31 ± 0,07	n.d.	n.d.	n.d.	1,02 ± 0,20
			Apr	0,17 ± 0,04	0,37 ± 0,08	0,78 ± 0,13	1,64 ± 0,14	0,26 ± 0,08	0,65 ± 0,16	0,45 ± 0,08	0,16 ± 0,09	0,26 ± 0,07	4,73 ± 0,50
8 (sea water)	River mouth 500m south	40°28′47”N 14°56′16″E	Jul	3,05 ± 0,24	6,02 ± 0,51	1,26 ± 0,25	11,78 ± 0,77	1,95 ± 0,24	4,92 ± 0,38	1,14 ± 0,15	3,89 ± 0,20	1,95 ± 0,29	35,96 ± 0,56
			Nov	0,61 ± 0,15	1,17 ± 0,18	1,06 ± 0,13	5,15 ± 0,15	1,85 ± 0,16	0,73 ± 0,15	0,80 ± 0,15	0,58 ± 0,10	1,85 ± 0,17	13,81 ± 0,04
			Feb	0,37 ± 0,09	0,95 ± 0,16	0,52 ± 0,08	3,56 ± 0,12	0,95 ± 0,12	1,66 ± 0,26	0,52 ± 0,16	0,20 ± 0,07	0,95 ± 0,18	9,67 ± 0,46
			Apr	0,76 ± 0,13	2,81 ± 0,18	2,40 ± 0,19	8,64 ± 0,41	0,92 ± 0,19	2,04 ± 0,19	1,21 ± 0,20	0,88 ± 0,22	0,92 ± 0,16	20,58 ± 1,42
9 (sea water)	River mouth 1000m south	40°28′39”N 14°55′56″E	Jul	2,61 ± 0,27	3,24 ± 0,18	0,43 ± 0,11	11,33 ± 0,57	0,99 ± 0,19	1,86 ± 0,19	0,47 ± 0,08	1,42 ± 0,18	0,99 ± 0,16	23,35 ± 1,43
			Nov	0,18 ± 0,08	0,84 ± 0,13	0,32 ± 0,05	3,03 ± 0,18	0,90 ± 0,17	0,57 ± 0,11	0,36 ± 0,07	0,12 ± 0,05	0,90 ± 0,16	7,21 ± 0,13
			Feb	0,16 ± 0,05	0,29 ± 0,06	0,22 ± 0,05	2,88 ± 0,23	0,34 ± 0,09	0,76 ± 0,12	0,47 ± 0,09	0,13 ± 0,04	0,43 ± 0,11	5,67 ± 0,27
			Apr	0,38 ± 0,11	1,22 ± 0,13	1,77 ± 0,29	5,75 ± 0,31	0,53 ± 0,18	1,14 ± 0,17	1,17 ± 0,17	0,30 ± 0,08	0,53 ± 0,12	12,79 ± 0,23
10 (sea water)	River mouth 1500m south	40°28′30”N 14°55′38″E	Jul	0,80 ± 0,08	0,77 ± 0,12	0,11 ± 0,03	5,30 ± 0,16	0,35 ± 0,06	0,30 ± 0,08	0,21 ± 0,05	0,58 ± 0,14	0,35 ± 0,10	8,78 ± 0,11
			Nov	0,14 ± 0,06	0,58 ± 0,10	0,30 ± 0,05	1,74 ± 0,15	0,40 ± 0,04	0,38 ± 0,07	n.d.	n.d.	0,41 ± 0,09	3,99 ± 0,21
			Feb	0,17 ± 0,03	0,29 ± 0,07	0,22 ± 0,06	1,11 ± 0,14	0,19 ± 0,04	0,27 ± 0,06	0,23 ± 0,06	n.d.	0,22 ± 0,06	2,71 ± 0,18
			Apr	0,32 ± 0,10	0,45 ± 0,12	0,89 ± 0,14	2,64 ± 0,14	0,41 ± 0,11	0,82 ± 0,15	0,61 ± 0,13	0,16 ± 0,06	0,41 ± 0,12	6,72 ± 0,93

n.d.: not detected.

Table S2. Description of the sampling sites and OPPs concentration (ng L^{-1}) with standard deviations (SD) detected in the suspended particulate matter (SPM) samples from the Sele River, southern Italy.

Sampling location			Sampling season	Organophosphate pesticides concentrations (ng L^{-1}) ± standard deviations (SD)										
Site number identification	Site characteristics	Site location	Campaigns	Diazinon	Dimethoate	Malathion	Clorpyrifos	Pirimiphos - Methyl	Fenitrothion	Methidathion	Tolclofos-Methyl	Parathion	Total (ng L^{-1})	Total (ng g^{-1} dw)
			Jul	1,63 ± 0,27	4,11 ± 0,16	1,40 ± 0,41	6,58 ± 0,37	1,54 ± 0,25	1,23 ± 0,24	0,44 ± 0,13	2,64 ± 0,33	0,80 ± 0,13	20,37 ± 0,23	1571,91 ± 47,81
1	Sele River	40°28′55”N	Nov	0,36 ± 0,13	0,84 ± 0,16	0,65 ± 0,14	1,30 ± 0,16	0,27 ± 0,10	n.d.	0,26 ± 0,10	0,49 ± 0,16	0,43 ± 0,12	4,59 ± 0,64	469,71 ± 75,12
(river water)	mouth	14°56′33″E	Feb	0,33 ± 0,11	0,43 ± 0,15	0,26 ± 0,06	0,81 ± 0,17	n.d.	n.d.	n.d.	n.d.	0,14 ± 0,07	1,96 ± 0,31	94,66 ± 14,38
			Apr	0,66 ± 0,18	1,43 ± 0,16	1,01 ± 0,11	2,04 ± 0,15	0,51 ± 0,09	0,50 ± 0,15	0,46 ± 0,08	0,51 ± 0,11	0,92 ± 0,14	8,04 ± 0,29	1025,00 ± 87,17
			Jul	1,75 ± 0,17	2,74 ± 0,23	0,36 ± 0,08	5,47 ± 0,18	1,24 ± 0,16	1,76 ± 0,17	0,63 ± 0,10	0,43 ± 0,08	0,25 ± 0,04	14,63 ± 0,84	1099,64 ± 31,80
2	River mouth	40°29′04”N	Nov	0,27 ± 0,09	0,85 ± 0,14	0,39 ± 0,17	1,29 ± 0,10	0,48 ± 0,11	0,29 ± 0,09	n.d.	n.d.	0,28 ± 0,11	3,84 ± 0,58	174,65 ± 28,29
(sea water)	500m north	14°56′14″E	Feb	n.d.	1,52 ± 0,20	n.d.	1,27 ± 0,13	n.d.	0,69 ± 0,18	n.d.	n.d.	n.d.	3,48 ± 0,25	34,08 ± 2,35
			Apr	0,38 ± 0,10	1,91 ± 0,18	0,67 ± 0,12	1,99 ± 0,16	0,40 ± 0,09	n.d.	0,72 ± 0,14	0,42 ± 0,06	0,46 ± 0,14	6,95 ± 0,75	816,88 ± 65,64
			Jul	1,33 ± 0,23	2,08 ± 0,25	n.d.	3,01 ± 0,23	0,78 ± 0,12	0,60 ± 0,17	0,24 ± 0,05	0,21 ± 0,07	n.d.	8,25 ± 0,16	670,59 ± 36,86
3	River mouth	40°29′12”N	Nov	n.d.	0,36 ± 0,11	n.d.	0,75 ± 0,13	n.d.	n.d.	n.d.	n.d.	n.d.	1,11 ± 0,24	109,04 ± 24,81
(sea water)	1000m north	14°55′56″E	Feb	n.d.	n.d.	n.d.	0,60 ± 0,10	n.d.	0,29 ± 0,04	n.d.	n.d.	n.d.	0,89 ± 0,08	9,64 ± 0,83
			Apr	n.d.	0,54 ± 0,08	0,83 ± 0,14	1,14 ± 0,15	n.d.	nd	0,36 ± 0,12	n.d.	0,74 ± 0,15	3,62 ± 0,47	581,61 ± 55,66
			Jul	0,34 ± 0,06	0,30 ± 0,07	n.d.	1,59 ± 0,14	0,25 ± 0,11	n.d.	n.d.	n.d.	n.d.	2,48 ± 0,07	174,19 ± 8,01
4	River mouth	40°29′20”N	Nov	n.d.	n.d.	n.d.	n.d.	n.d.	n.d.	n.d.	n.d.	n.d.	n.d.	n.d.
(sea water)	1500m north	14°55′38″E	Feb	n.d.	n.d.	n.d.	n.d.	n.d.	n.d.	n.d.	n.d.	n.d.	n.d.	n.d.
			Apr	n.d.	n.d.	0,23 ± 0,06	0,50 ± 0,15	n.d.	n.d.	0,23 ± 0,07	n.d.	0,13 ± 0,06	1,08 ± 0,12	123,84 ± 8,85
			Jul	1,06 ± 0,17	2,26 ± 0,17	n.d.	4,14 ± 0,25	0,49 ± 0,12	0,83 ± 0,12	n.d.	0,28 ± 0,06	n.d.	9,06 ± 0,34	1120,17 ± 67,32
5	River mouth	40°28′55”N	Nov	n.d.	n.d.	n.d.	n.d.	n.d.	n.d.	n.d.	n.d.	n.d.	n.d.	n.d.
(sea water)	500m west	14°56′12″E	Feb	n.d.	n.d.	n.d.	n.d.	n.d.	n.d.	n.d.	n.d.	n.d.	n.d.	n.d.
			Apr	0,53 ± 0,13	0,28 ± 0,07	1,17 ± 0,12	2,03 ± 0,11	0,22 ± 0,05	n.d.	0,26 ± 0,09	0,30 ± 0,09	0,97 ± 0,11	5,77 ± 0,31	537,07 ± 44,31
			Jul	n.d.	n.d.	n.d.	n.d.	n.d.	n.d.	n.d.	n.d.	n.d.	n.d.	n.d.
6	River mouth	40°28′55”N	Nov	n.d.	n.d.	n.d.	n.d.	n.d.	n.d.	n.d.	n.d.	n.d.	n.d.	n.d.
(sea water)	1000m west	14°55′50″E	Feb	n.d.	n.d.	n.d.	n.d.	n.d.	n.d.	n.d.	n.d.	n.d.	n.d.	n.d.
			Apr	n.d.	n.d.	n.d.	n.d.	n.d.	n.d.	n.d.	n.d.	n.d.	n.d.	n.d.
				n.d.	n.d.	n.d.	n.d.	n.d.	n.d.	n.d.	n.d.	n.d.	n.d.	n.d.
			Jul	n.d.	n.d.	n.d.	n.d.	n.d.	n.d.	n.d.	n.d.	n.d.	n.d.	n.d.
7	River mouth	40°28′55”N	Nov	n.d.	n.d.	n.d.	n.d.	n.d.	n.d.	n.d.	n.d.	n.d.	n.d.	n.d.
(sea water)	1500m west	14°55′28″E	Feb	n.d.	n.d.	n.d.	n.d.	n.d.	n.d.	n.d.	n.d.	n.d.	n.d.	n.d.
			Apr	n.d.	n.d.	n.d.	n.d.	n.d.	n.d.	n.d.	n.d.	n.d.	n.d.	n.d.
			Jul	1,32 ± 0,14	2,81 ± 0,22	n.d.	5,57 ± 0,17	0,78 ± 0,15	2,38 ± 0,15	0,58 ± 0,10	2,18 ± 0,26	n.d.	15,62 ± 0,92	1442,66 ± 95,72
8	River mouth	40°28′47”N	Nov	n.d.	0,57 ± 0,11	0,50 ± 0,10	2,44 ± 0,12	0,66 ± 0,13	0,28 ± 0,10	0,39 ± 0,08	n.d.	0,41 ± 0,13	5,25 ± 0,47	245,72 ± 18,86
(sea water)	500m south	14°56′16″E	Feb	n.d.	n.d.	n.d.	2,44 ± 0,20	n.d.	0,80 ± 0,12	n.d.	n.d.	n.d.	3,24 ± 0,31	34,06 ± 3,38
			Apr	n.d.	1,23 ± 0,16	1,08 ± 0,16	4,21 ± 0,14	0,37 ± 0,12	n.d.	0,63 ± 0,13	0,33 ± 0,06	0,88 ± 0,13	8,73 ± 0,24	946,05 ± 13,24
			Jul	1,26 ± 0,13	0,84 ± 0,17	n.d.	5,98 ± 0,37	n.d.	0,93 ± 0,13	n.d.	0,62 ± 0,17	n.d.	9,78 ± 0,08	693,80 ± 15,44
9	River mouth	40°28′39”N	Nov	n.d.	0,39 ± 0,08	n.d.	1,20 ± 0,11	0,40 ± 0,16	n.d.	n.d.	n.d.	n.d.	1,99 ± 0,17	101,89 ± 9,00
(sea water)	1000m south	14°55′56″E	Feb	n.d.	n.d.	n.d.	1,36 ± 0,17	n.d.	n.d.	n.d.	n.d.	n.d.	1,36 ± 0,17	13,58 ± 1,69
			Apr	n.d.	n.d.	0,50 ± 0,12	2,50 ± 0,20	n.d.	n.d.	0,62 ± 0,15	n.d.	0,40 ± 0,10	4,02 ± 0,18	622,66 ± 36,86
			Jul	n.d.	n.d.	n.d.	2,64 ± 0,13	n.d.	n.d.	n.d.	n.d.	n.d.	2,64 ± 0,13	239,55 ± 1,15
10	River mouth	40°28′30”N	Nov	n.d.	n.d.	n.d.	0,68 ± 0,17	n.d.	n.d.	n.d.	n.d.	n.d.	0,68 ± 0,17	23,47 ± 6,21
(sea water)	1500m south	14°55′38″E	Feb	n.d.	n.d.	n.d.	n.d.	n.d.	n.d.	n.d.	n.d.	n.d.	n.d.	n.d.
			Apr	n.d.	n.d.	n.d.	n.d.	n.d.	n.d.	n.d.	n.d.	n.d.	n.d.	n.d.

n.d.: not detected.
